# Supplementary material for: Subtomogram averaging of COPII assemblies reveals how coat organization dictates membrane shape
Source: Nat Commun. 2018 Oct 8;9:4154. doi: 10.1038/s41467-018-06577-4 (PMC6175875; doi:10.1038/s41467-018-06577-4)
Supplement: Supplementary file 1 — Supplementary Information [file 41467_2018_6577_MOESM1_ESM.pdf]

# Sub-tomogram averaging of COPII assemblies reveals how coat organization dictates membrane shape.

J. Hutchings *et al*.

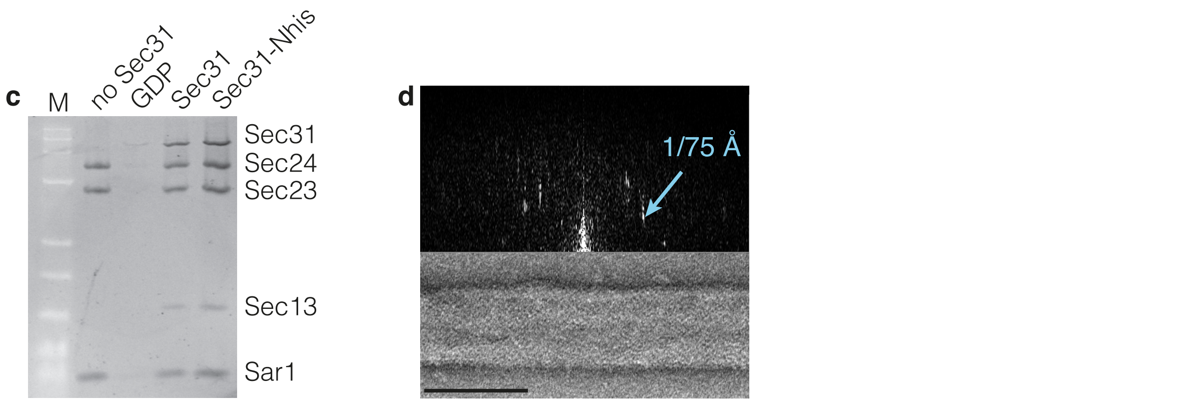


**Supplementary Figure 1. Inner coat assembly depends on outer coat binding.**

**(a)** Bottom: a negative stain image of a budding reaction with wild type Sec13-Sec31 (N-his tag cleaved), showing the characteristic outer coat pattern. Top: zoomed power spectrum, with peaks consistent with reported outer coat spacings. Scale bar 50 nm.

**(b)** Negative stain images of budding reconstituted without Sec13/31. Left: tubular regions of membranes are less rigid. Right: even straighter tubules show no evidence of ordered inner coat, as shown by the absence of any peaks on the power spectrum. Scale bars: 500 nm (left) and 50 nm (right).

**(c)** COPII coat recruitment to small unilamellar vesicles was measured following flotation through a sucrose gradient. Sar1, Sec23-Sec24 and Sec13-Sec31 were incubated with liposomes and GMP-PNP as indicated, floated liposomes recovered and bound proteins detected by SYPRO stain. M=molecular weight markers; positions of COPII proteins are indicated.

**(d)** Bottom: a negative stain image of the same reaction as in Fig 1B, with zoomed power spectrum, showing a pattern characteristic of the inner coat, but no outer coat. Scale bar: 50nm.

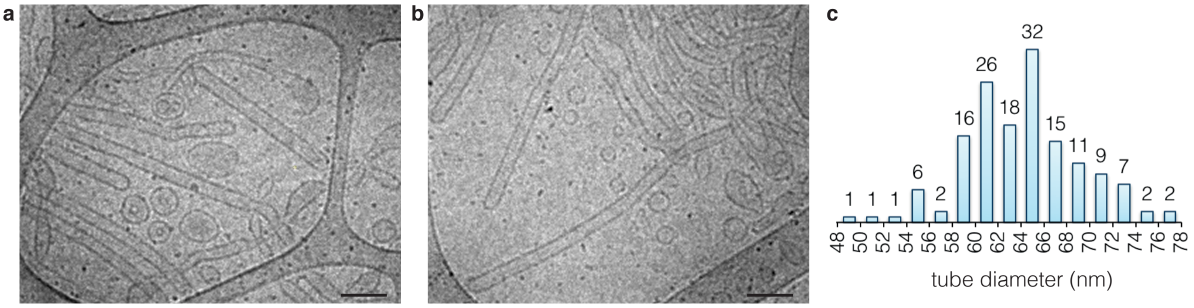


**Supplementary Figure 2. Overview of budding morphologies**

**(a-b)** Representative images showing overviews of reconstituted budding using N-his Sec31, indicating the presence of straight tubules of various diameters, as well as heterogeneous vesicles and more irregular membranes. Scale bars 100nm. These images are from the data collection that generated the subtomogram average.

**(c)** Distribution of diameters of COPII tubules, measured on the dataset used for sub-tomogram averaging.

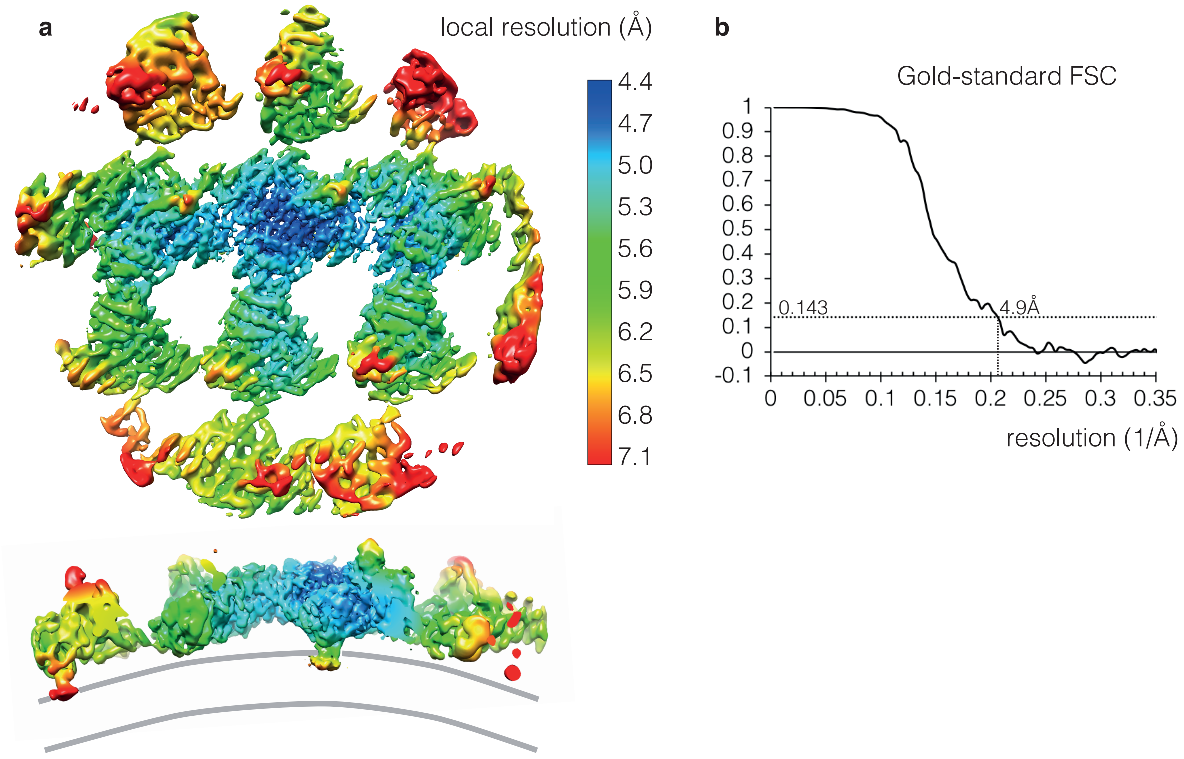


**Supplementary Figure 3. Resolution estimates**

**(a)** Local resolution estimated with Relion LocRes.

**(b)** Mask-corrected gold standard Fourier Shell Correlation.

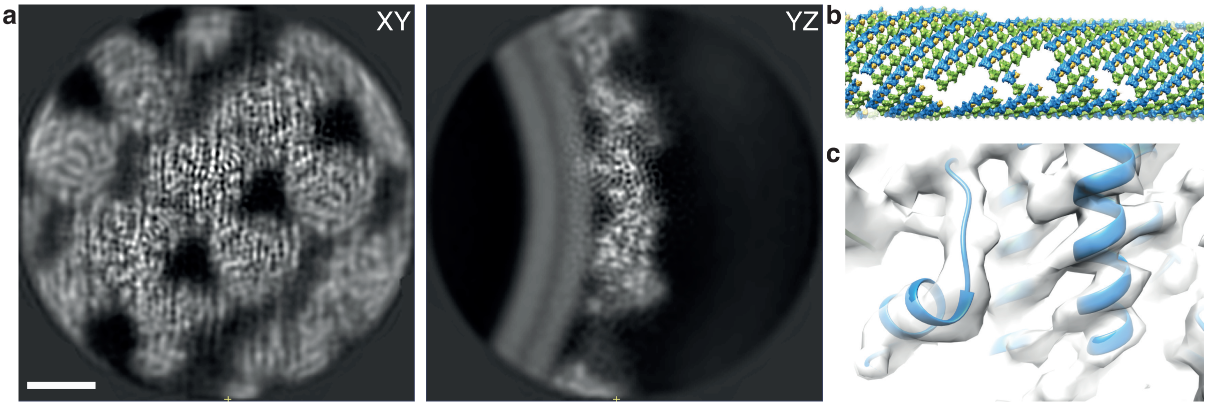


**Supplementary Figure 4. Views of the subtomogram averaging structure**

**(a)** Slices through XY and YZ planes of the sub-tomogram average map.

**(b)** Averages are back-plotted as in Figure 2A to show a tube coated in patches, which changes diameter.

**(c)** A detail of one of the highest resolution regions of Sec23.

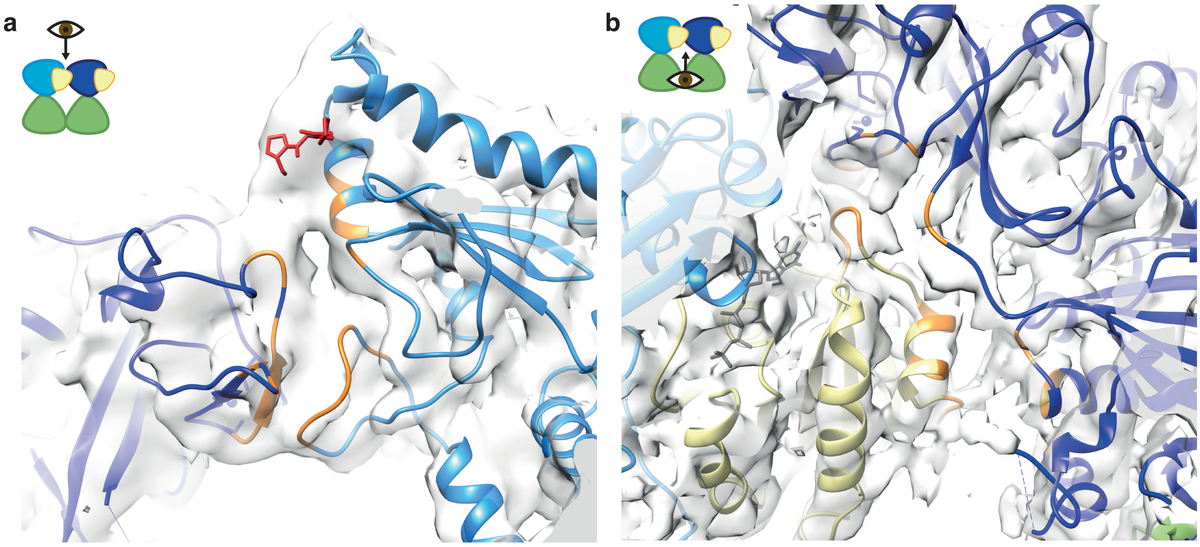


**Supplementary Figure 5. Inner coat lattice interfaces**

**(a)** View of the Sec23-Sec23 interface, with contacting residues identified by PISA colored in pink.

**(b)** View of the inter-subunit Sar1-Sec23 interface, with contacting residues identified by PISA colored in pink.

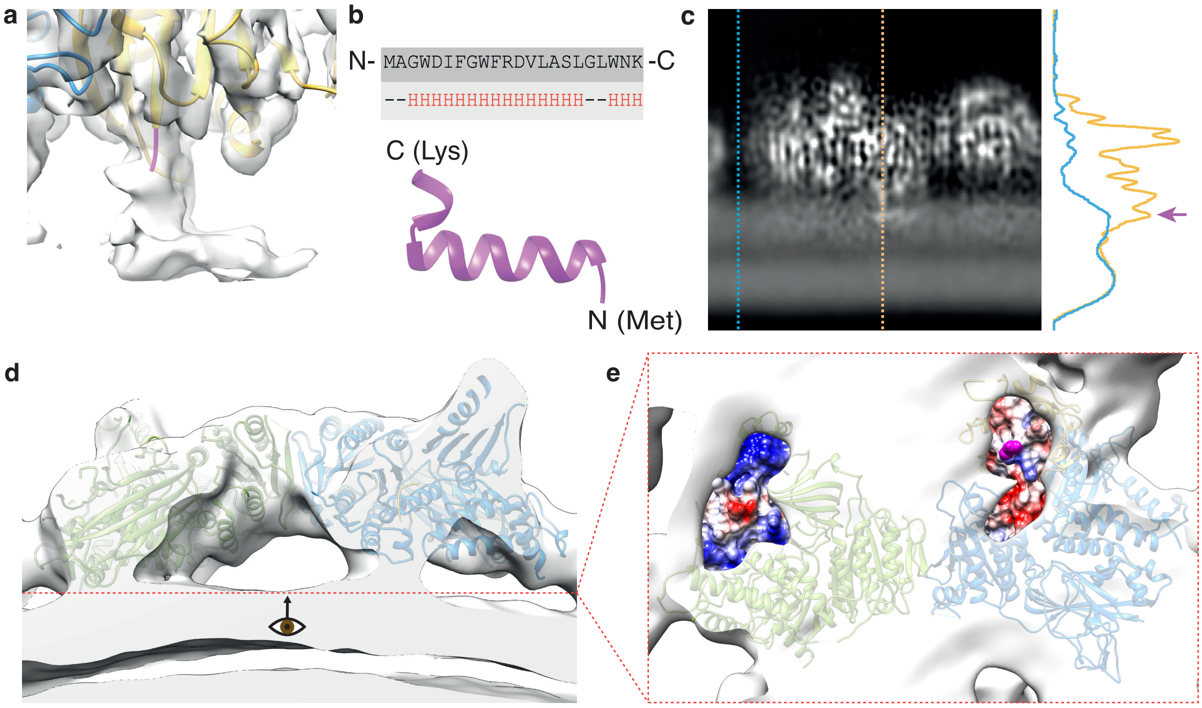


**Supplementary Figure 6. Interactions with the membrane**

**(a)** View of the Sar1 N-terminal amphipathic helix density

**(b)** Secondary structure prediction shows an interruption of the helix at residues G18-L19, which was modelled as a kink by the structure prediction software Robetta. The modelled helix corresponds well to the density.

**(c)** Orthoslice view of the average structure, showing the region of membrane insertion. Density profiles through the density as indicated by the dotted lines are shown with corresponding colours.

**(d)** Side view of the gaussian-filtered subtomogram average with the fitted model to show Sec23 and Sec24 intimately contact the membrane.

**(e)** Bottom view from inside the outer membrane leaflet of (c). The regions of the model that are in direct contact with the membrane are represented as surfaces coloured according to their Coulombic potential, as calculated using UCSF Chimera.

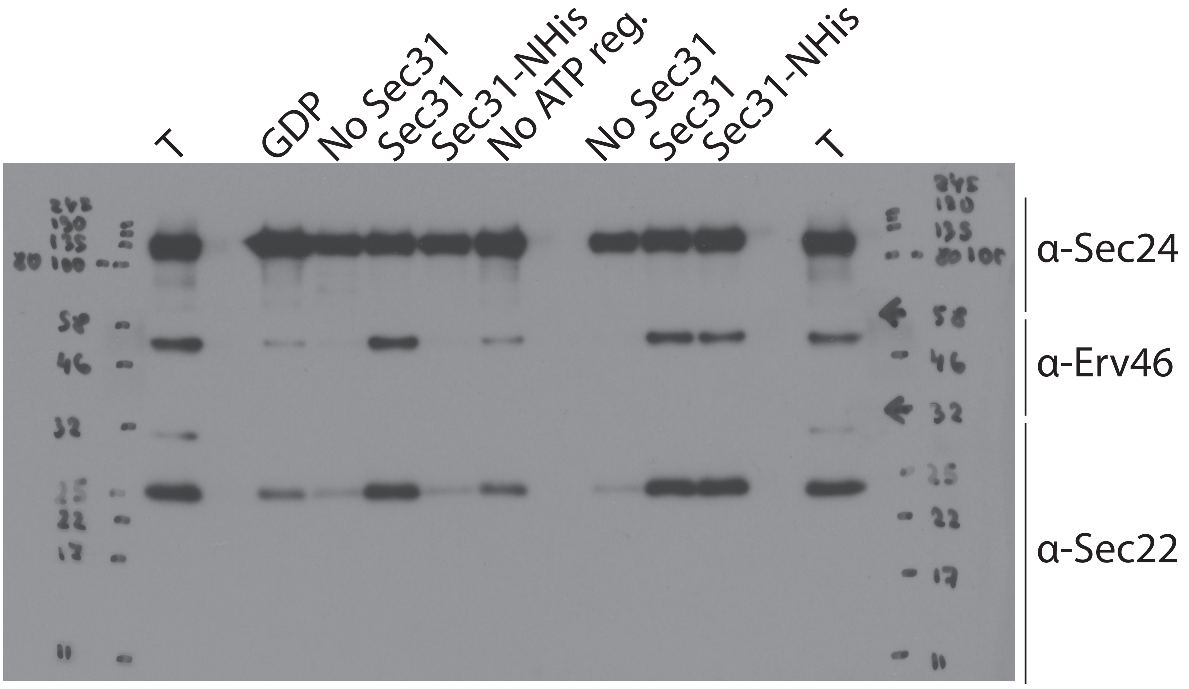


**Supplementary Figure 7. Immunoblotting of *in vitro* budding reactions with microsomal membranes**

The original image used in Figure 1D. Marker lanes (Color Prestain Protein Standard, Broad Range, NEB) are indicated on the left and right hand side of the blot. Arrows on the right hand side indicate where the gel was cut for incubation with the relevant antibody labeled on the right. T corresponds to 10% of the total donor membranes, other lanes refer to vesicles.

| Primer name | Sequence (5’ – 3’) | Function |
|---|---|---|
| **Sec31-fo** | CTGTATTTTCAGGGC<br>ATGGTCAAACTTGCTGAGTTTTCTC | In-Fusion with pFASTbacHTb-re (includes HIS and TEV) |
| **Sec31-re** | CTTGGTACCGCATGC<br>*TTA*ATTCAAAGTCGCTTCAGCTATG | In-Fusion with pFASTbacHTb-fo. *TTA* is stop |
| **Sec13-fo** | CGGTCCGAAACCATG<br>ATGGTCGTCATAGCTAATGCGC | In-Fusion with pFASTbacHTb-notag-re (no HIS and TEV). |
| **Sec13-re** | CTTGGTACCGCATGC<br>*TCA*CTGATGAACTTCACCAGCG | In-Fusion with pFASTbacHTb-fo. *TCA* is stop. |
| **Sec24-fo** | CTGTATTTTCAGGGC<br>ATGTCTCATCACAAGAAACGTGTTTAC | In-Fusion with pFASTbacHTb-re (includes HIS and TEV) |
| **Sec24-re** | CTTGGTACCGCATGC<br>*TTA*TTTGCTAATTCTGGCTTTCATG | In-Fusion with pFASTbacHTb-fo. *TTA* is stop. |
| **Sec23-fo** | CGGTCCGAAACCATG<br>ATGGACTTCGAGACTAATGAAGACATC | In-Fusion with pFASTbacHTb-notag-re (no HIS or TEV). |
| **Sec23-re** | CTTGGTACCGCATGC<br>*CTA*TGCCTGACCAGAGACGG | In-Fusion with pFASTbacHTb-fo. *CTA* is stop. |
| **pFASTbacHTb-re** | GCCCTGAAAATACAGGTTTTCGGTC | Reverse primer to amplify pFASTBacHT-b up to HIS and TEV cleavage site. |
| **pFASTbacHTb-notag-re** | CATGGTTTCGGACCGAGATCCG | Reverse primer to amplify pFASTBacHT-b (no HIS and TEV). |
| **pFASTbacHTb-fo** | GCATGCGGTACCAAGCTTGTC | Forward primer to amplify pFASTBacHT-b. |
| **Sar1-fo** | CTTTATTTTCAGGGC<br>ATGGCTGGTTGGGATATTTTTG | In-Fusion with petm11-re. |
| **Sar1-re** | ATCCGGTACCACTAG<br>*TTA*AATATATTGAGATAACCATTGGAACG | In-Fusion with petm11-fo. *TTA* is stop. |

| | | |
|---|---|---|
| **Sar1-intr-fo** | GGTTGGT TCAGAGATGTGTTGGCTTCCC | Amplify Sar1 insert before intron + 7 sticky bases after intron. |
| **Sar1-intr-re** | ATCTCTGA ACCAACCAAAAATATCCCAACC | Amplify Sar1 insert after intron + 8 sticky bases before intron. |
| **petm11-fo** | CTAGTGGTACCGGATCCGAATTC | Forward primer to amplify pETM-11. |
| **petm11-re** | GCCCTGAAAATAAAGATTCTCAGTAGTG | Reverse primer to amplify pETM-11. |

**Supplementary Table 1. Primer sequences used in this study**

The primer name, sequence and a brief description of its use is provided. Underlined parts of sequences indicate complementary overhangs for In-Fusion cloning (Takara) with either pETM-11 for Sar1 or pFASTbacHTb for Sec23/24 and Sec13/31. Italics indicate a STOP codon.

| | #1 name (EMDB-0044) (PDB 6gni) |
|---|---|
| **Data collection and processing** | |
| Magnification | EFTEM 105k |
| Voltage (kV) | 300 |
| Electron exposure (e–/Å$^2$) | ~ 147 |
| Defocus range (μm) | 1.5 – 3.5 |
| Pixel size (Å) | 1.327 |
| Symmetry imposed | C1 |
| Initial particle images (no.) | ~ 400,000 |
| Final particle images (no.) | 87,952 |
| Map resolution (Å) | 4.87 |
| FSC threshold | 0.143 |
| Map resolution range (Å) | 4.4 - 7 |
| | |
| **Refinement** | |
| Initial model used (PDB code) | 1m2o and 1m2v |
| Model resolution (Å) | 4.9 |
| FSC threshold | (0.143) |
| Model resolution range (Å) | 4.9 |
| Map sharpening *B* factor (Å$^2$) | -350 |
| Model composition | |
| Non-hydrogen atoms | 12937 |
| Protein residues | 1632 |
| Ligands | 4 |
| R.m.s. deviations | |
| Bond lengths (Å) | 0.38 |
| Bond angles (°) | 0.65 |
| Validation | |
| Clashscore | 20 |
| Poor rotamers (%) | 3.5 |
| Ramachandran plot | |
| Favored (%) | 91 |
| Allowed (%) | 6 |
| Disallowed (%) | 2 |

**Supplementary Table 2. Cryo-EM data collection, refinement and validation statistics**
