# Supplementary material for: Subtomogram averaging of COPII assemblies reveals how coat organization dictates membrane shape
Source: Nat Commun. 2018 Oct 8;9:4154. doi: 10.1038/s41467-018-06577-4 (PMC6175875; doi:10.1038/s41467-018-06577-4)
Supplement: Supplementary file 3 — Description of Additional Supplementary Files [file 41467_2018_6577_MOESM3_ESM.pdf]

Description of Additional Supplementary Files

File Name: Supplementary Movie 1
Description: An overview of the COPII inner coat assembled on membranes, with a summary of the main results from this study.
